# Antimicrobial Nanoemulsion Formulation with Improved Penetration of Foliar Spray through Citrus Leaf Cuticles to Control Citrus Huanglongbing

**DOI:** 10.1371/journal.pone.0133826

**Published:** 2015-07-24

**Authors:** Chuanyu Yang, Charles A. Powell, Yongping Duan, Robert Shatters, Muqing Zhang

**Affiliations:** 1 College of Crop Science, Fujian Agriculture and Forestry University, Fuzhou, China; 2 Indian River Research and Education Center, IFAS, University of Florida, Fort Pierce, FL, United States of America; 3 Horticultural Research Lab, USDA-ARS, Fort Pierce, FL, United States of America; 4 State Key Lab for Conservation and Utilization of Subtropical Agri-biological Resources, Guangxi University, Nanning, China; Ghent University, BELGIUM

## Abstract

Huanglongbing (HLB) is the most serious disease affecting the citrus industry worldwide to date. The causal agent, *Candidatus *Liberibacter asiaticus (Las), resides in citrus phloem, which makes it difficult to effectively treat with chemical compounds. In this study, a transcuticular nanoemulsion formulation was developed to enhance the permeation of an effective antimicrobial compound (ampicillin; Amp) against HLB disease through the citrus cuticle into the phloem via a foliar spray. The results demonstrated that efficiency of cuticle isolation using an enzymatic method (pectinase and cellulase) was dependent on the citrus cultivar and Las-infection, and it was more difficult to isolate cuticles from valencia orange (*Citrus sinensis*) and HLB-symptomatic leaves. Of eight adjuvants tested, Brij 35 provided the greatest increase in permeability of the HLB-affected cuticle with a 3.33-fold enhancement of cuticular permeability over water control. An *in vitro *assay using *Bacillus subtilis* showed that nanoemulsion formulations containing Amp (droplets size = 5.26 ± 0.04 nm and 94 ± 1.48 nm) coupled with Brij 35 resulted in greater inhibitory zone diameters (5.75 mm and 6.66 mm) compared to those of Brij 35 (4.34 mm) and Amp solution (2.83 mm) alone. Furthermore, the nanoemulsion formulations eliminated Las bacteria in HLB-affected citrus *in planta* more efficiently than controls. Our study shows that a water in oil (W/O) nanoemulsion formulation may provide a useful model for the effective delivery of chemical compounds into citrus phloem via a foliar spray for controlling citrus HLB.

## Introduction

The genus *Citrus* comprises a group of economically important fruits. Although prone to a number of diseases, huanglongbing (HLB) is the most destructive disease threatening the citrus industry worldwide. The disease is caused by three species of gram-negative and phloem-restricted *Candidatus* Liberibacter spp., which can be transmitted by *Diaphorina citri* or *Trioza erytreae* [1–5]. HLB disease caused costs to the economy of Florida of an estimated $3.63 billion in lost revenue as well as 6,611 lost jobs by reducing orange juice production since 2006 [6].

The HLB disease is difficult to control in the field due to lack of HLB-resistant cultivars, the rapid rate of disease spreading, and the fastidious nature of the pathogens [7, 8]. In our previous studies, we screened for compounds with antimicrobial activity against citrus HLB [9–12], such as ampicillin, carbenicillin, and penicillin. However, no compounds to date have been used commercially to control HLB. In order to be efficacious for controlling citrus HLB, the antimicrobial compounds should have the following characteristics: (i) be active inside of the plant; (ii) have efficient delivery to the phloem; (iii) be tolerant of oxidation, UV irradiation, rainfall, and high temperatures; (iv) be non-phytotoxic to citrus; and (v) have a low or non-detectable rate of resistant pathogens. Due to uncultured Las residing in phloem as well as the barrier of the plant cuticle, it has been difficult to identify and deliver effective compounds against Las into the phloem through the citrus leaf cuticle or bark using a foliar spray or bark painting.

The plant cuticle is composed of three distinct substances (*i*.*e*., waxes, cutin, and pectin) that typically act as barriers against the entry of many compounds into plant tissues [13, 14]. Several studies have demonstrated that the structure and composition of the cuticle are different among citrus species and cultivars and that these differences are reflected in cuticular permeability as well as the susceptibility of citrus leaves to pathogen infection [15–17]. Certain chemical adjuvants (penetration enhancing components) can directly modify leaf waxes and increase film tension of the leaf surface in order to enhance cuticular permeability [18, 19]. Foliar spray of adjuvants onto citrus has been associated with increased nutrition and effective fungicidal activity [20–22]. However, few studies have been conducted on the application of adjuvants by foliar spray with the aim of controlling HLB, and limited results have demonstrated that adjuvants such as dimethyl sulfoxide (DMSO) and Silwet L-77 have no significant effect on treatment when combined with Penicillin and Streptomycin (PS) in HLB-affected citrus [11]. Therefore, screening candidate adjuvants will help to enhance citrus cuticular permeability for controlling the HLB disease.

Nanoemulsion is a useful technology for delivering chemical compounds across the cuticle. The efficacy of this approach is based on the small size of nanoemulsion droplets, the large surface area of the emulsion, the low surface tension of the entire system, and the low interfacial tension of the droplets [23]. To date, there have been several reports on the control of plant disease using nanotechnology [24, 25], but there is as yet no evidence concerning the applicability of nanoemulsion for controlling HLB disease. We hypothesized that a nanoemulsion technology combined with a suitable adjuvant for enhancing permeability may be able to effectively deliver antibacterial compounds into citrus phloem in order to combat the HLB bacterium Las. In this report, we screened a number of candidate adjuvants for their efficacy on the HLB-affected cuticle and optimized a nanoemulsion for delivering effective antibacterial compounds into the citrus phloem by foliar spray.

## Materials and Methods

### Plant materials

Two-year-old seedlings from lemon (*Citrus limon*), grapefruit (*Citrus paradisi*), bitter orange (*Citrus aurantium*), and valencia orange (*Citrus sinensis*) were planted from Las-free seeds and kept in the HLB-free greenhouse at USHRL, USDA-ARS (Fort Pierce, Florida). The seedlings from grapefruit (*Citrus paradisi*) were graft-inoculated with HLB-affected lemon (*Citrus limon*) scions and were subsequently maintained in an insect-proof greenhouse. After ten months, typical HLB symptoms, including vein corking and blotchy mottles, appeared on the leaves of inoculated grapefruit seedlings. HLB-affected citrus seedlings with typical HLB symptoms were tested for the presence of Las bacteria using quantitative real-time PCR (qPCR) with Las-specific primers (HLBas, HLBr, and HLBp) [26].

### Cuticle isolation for screening adjuvants of enhancing penetration

A split-split plot experimental design [27, 28] was used to optimize cuticle isolation from four Las-free citrus cultivars, *i*.*e*. lemon (*Citrus limon*), grapefruit (*Citrus paradisi*), bitter orange (*Citrus aurantium*), and valencia orange (*Citrus sinensis*), in the HLB-free greenhouse using Schönherr’s method with some modifications [29]. The entire plot consisted of citrus cultivar (lemon, grapefruit, bitter orange, and valencia orange), while leaf disk size (10 mm, 7 mm, and 5 mm in diameter) and the concentrations of pectinase and cellulase in the isolation buffer, *i*.*e*. (i) 4% pectinase and 0.4% cellulase, (ii) 2% pectinase and 0.2% cellulose, and (iii) 1% pectinase and 0.1% cellulase, were considered as the split and sub-split plots, respectively. Briefly, leaf disks were first punched out from citrus leaves and then incubated in 12 ml of citrate buffer (50 mM, pH 4.0) containing pectinase (Pfaltz&Bauer, pectinase from *Aspergillis niger* > 1.0 unit/mg) and cellulase (MP Biomedicals, LLC) in 50 ml disposable tubes at room temperature. When the cuticle in each tube was completely separated from the leaf disks, the incubation time was recorded.

### Transcuticular movement bioassay and its characteristics of the cuticle from HLB-affected citrus leaves

The leaves from symptomatic and asymptomatic leaves of HLB-affected lemon (*Citrus limon*) citrus were collected for cuticle isolation and assessed for sucrose and pectinase activity. A 7 mm section of each cuticle was isolated from the leaf disks and incubated in 2% pectinase and 0.2% cellulase.

#### Transcuticular movement bioassay

Transcuticular movement was assayed *in vitro* as described by Solel and Edgington (1973) [30]. The ampicillin-sensitive bacterium *Bacillus subtilis* was spread onto LB agar medium in petri dishes before application of the chemical treatment. Cuticle disks were placed on the surface of inoculated petri dishes and the indicated chemicals were applied to the cuticles in 5 μl droplets. Dished were then incubated at 28°C for 24 h. Following this incubation period, the inhibition zone of bacterial growth was measured. The zone diameter was indicative of the amount of chemical that moved through the isolated cuticle during the experimental period.

#### Sucrose assay

Leaf samples were frozen and ground in liquid nitrogen. Sucrose was extracted using 80% (v/v) ethanol (Fisher Scientific, Pittsburgh, PA) using a method previously described with minor modifications [31]. Briefly, 0.07 g of pulverized leaf material was submerged in 1.5 ml of ethanol and incubated at 60°C for 2.5 h with frequent mixing of the plant material by inverting the tubes 6 times. The tubes were then centrifuged at 14,000 rpm for 1 min and the supernatant was collected. The extraction steps were repeated three times. The pooled extract (7 ml) was treated with activated charcoal (Sigma-Aldrich, St. Louis, MO) to absorb phenols and chlorophylls that might interfere with enzymes for sucrose analysis. The sucrose assay was conducted according to Roe’s method [32]. A mixture of 100 μl of 2 M NaOH and 200 μl of extracted sucrose solution was incubated in boiling water for 5 min and then 1.3 ml of 30% HCl and 400 μl of 0.1% resorcinol (dissolved in alcohol) were added to stop the reaction. After cooling, the developed color was measured at 450 nm using a spectrophotometer (Beckman Coulter, Inc., Indianapolis IN) and the sucrose content was assayed in triplicate.

#### Pectinase assay

Four grams of citrus leaf were ground in a mortar with 20 ml distilled water and the slurry was centrifuged for 15 min at 3,000 rpm. Ten drops of toluene were added to the supernatant as a preservative for the pectinase assay [33]. Pectinase activity was assayed by the colorimetric method of Miller (1959) [34]. The reaction mixture contained 100 μl of 1% pectin prepared in 0.05 M citrate buffer (pH 4.0), 1.2 ml of 0.05 M citrate buffer (pH 4.0), 100 μl of leaf extract or 0.1% sucrose in 0.05 M citrate buffer (pH 4.0), and 100 μl of 0.2% pectinase. Assays were incubated at 50°C for 30 min in a water bath. No substrate (1% pectin only) in the reaction buffer was used for the blank control. Following incubation, 500 μl of 3, 5-dinitrosalicylic acid (DNS) reagent was added to stop the reaction and assay tubes were placed in boiling water for 10 min. After cooling, the developed color was measured at 540 nm using a spectrophotometer (Beckman Coulter, Inc., Indianapolis IN). Enzyme activity was averaged from assays performed in triplicate.

### Screening and evaluation of the candidate adjuvants for enhancing penetration

Eight adjuvants were evaluated by cuticular permeability, of which Brij 35 (CAS 9002-92-0), Brij 58 (CAS 9004-95-9), Brij 98 (CAS 9004-98-2), and Urea (CAS 57-13-6) were purchased from ACROS Organic (Thermo Fisher Scientific, NJ), dimethyl sulfoxide (DMSO, CAS 67-68-5) and 2-methyl-2-pyrrolidinone (NMP, CAS 872-50-4) from Sigma-Aldrich (St. Louis, MO), Laurocapram (CAS 59227-89-3) from NetQem LLC (Research Triangle Park, NC), and Oleic acid (CAS 112-80-1) from MP Biomedicals LLC (Santa Ana, CA).

Cuticular permeability was measured using transport chambers made of stainless steel as described by Schreiber [35]. Briefly, the isolated cuticle was mounted between donor and receiver compartments of the transport chamber and the cuticle/steel interface was sealed with high-vacuum silicon grease (Sigma-Aldrich, St. Louis, MO). Cuticles were oriented in such a way that the physiological outer side faced the donor compartment. Subsequently, 2 ml of 0.01 M borate buffer (pH 9.0, containing 0.1% adjuvant) was added to the receiver compartment of a transport chamber and 200 μl of 4-Nitrophenol (4-NP) (Thermo Fisher Scientific, NJ) solution in 0.01 M citric buffer (pH 3.0, containing 0.015 M of 4-NP) was added to the donor compartment of the transport chamber at time zero. Loaded transport chambers were incubated in the dark on a rotating bench (60 rpm) in a thermo-stable environment. The sample present in receiver cells was measured after incubation times of 0, 15 min, 30 min, 45 min, and 60 min at 399 nm by spectrophotometry (Beckman, Indianapolis IN).

Permeability of 4-NP (Permeability) was calculated according to the following equation:

Permeability=F/cA

Where F (mol.min^-1^) was the slope of the linear regression line fitted to the transport; c (mol.cm^-2^) was the concentration of 4-NP in the donor compartment, and A (cm^2^) was the area of the cuticle exposed in the transport chamber. The permeability for each treatment were averaged from 10 cuticles.

### Preparation and application of water in oil (W/O) nano-formulations

#### Preparation of W/O nano-formulations (Nano-1-Amp and Nano-2-Amp)

The methodology for obtaining W/O nano-formulations used the following three steps: (i) Preparation of nanoemulsion (Nano-1 and Nano-2, see Table 1). Homogeneous organic solution (S_1_), composed of 5.0625 g of Cremophor EL (Sigma-Aldrich, St. Louis, MO) and 0.1785 g of Span 80 (Sigma-Aldrich, St. Louis, MO) in 11.125 ml of Ethyl acetate (Thermo Fisher Scientific, NJ), was injected into the homogeneous aqueous phase (S_2_) composed of 0.03 g of Tween 80 (Sigma-Aldrich, St. Louis, MO) and 2.823 ml of tap water for Nano-1 (Table 1), or was formed from 0.03 g of Tween 80 and 0.125 g of ampicillin (Thermo Fisher Scientific, NJ) in 2.823 ml of tap water for Nano-2 (Table 1), by ultrasonic stirring for 5 min to reach equilibrium. Ethyl acetate was then removed by evaporation in a ventilated hood overnight. (ii) Two prepared nanoemulsions (Nano-1 and Nano-2) in a volume of 250 ml were diluted in the Amp solution (contained 0.125 g of Amp) for Nano-1 and tap water for Nano-2, respectively. Therefore, two nano-formulations (Nano-1-Amp and Nano-2-Amp) were formed at the same concentration of Amp (500 mg/L) in this step (Table 1). (iii) In order to obtain the novel nano-formulations (Nano-1-Amp+Brij 35, and Nano-2-Amp+Brij 35) for foliar spray, the screened adjuvant Brij 35 at a concentration of 0.1% (w/w) was added to Nano-1-Amp and Nano-2-Amp, respectively. Brij 35 was also added in the 500 mg/L ampicillin solution (Amp) and water (CK) as controls.

**Table 1:** Compositions and their percentage (%) of nanoemulsions (Nano-1-Amp and Nano-2-Amp).

Composition		Nano-1-Amp		Nano-2-Amp	
Nanoemulsion		Nano-1		Nano-2	
		Before evaporation(%)	After evaporation(%)	Before evaporation(%)	After evaporation(%)
**Organic phase(S**_**1**_ **)**	**Cremophor EL**	28.01	62.55	27.82	61.60
	**Span 80**	0.99	2.21	0.98	2.17
	**Ethyl acetate**	55.22	0.00	54.84	0.00
**Aqueous phase(S**_**2**_ **)**	**Tween 80**	0.17	0.37	0.16	0.37
	**water**	15.62	34.88	15.51	34.35
	**Amp**	0.00	0.00	0.69	1.52
**Diluent (after evaporation)**		Nano-1: Diluent(w/v^f^ ^)^		Nano-2: Diluent(w/v^f^ ^)^	
**500 mg/L Amp solution**		1:30.89		–	
**Tap water**		–		1:30.42	
**Final Amp concentration (mg/L)**		500		500	

^f^ w/v indicates the weight of nanoemulsion (g)/volume of diluent (ml).

#### Physicochemical characterizations of W/O nano formulations

Physicochemical analysis of nanoemulsions (Nano-1-Amp and Nano-2-Amp) was carried out immediately after preparation, including measurements of droplet size, thermodynamic stability, and pH value. The emulsion droplet size was determined by dynamic light scattering using Nanotrac 250 (Microtrac Inc. Montgomeryville, PA). Emulsion droplet size was estimated by the average of three measurements and presented as a mean diameter of the volume distribution (MV). Thermodynamic stability tests were performed by centrifugation after the freeze and thaw cycle. The prepared nanoemulsions were centrifuged at 3,500 rpm for 30 min. Nano-formulations without phase separation were taken for six heating and cooling cycles at temperatures of 4°C and 45°C for 48 h. Finally, the temperature-stable nanoemulsions were subjected to three freeze-thaw cycles between -21°C and +25°C. The pH value of the nanoemulsion was measured by immersing the pH electrode into emulsion dispersion using a pH meter (Fisher science education US) at 25°C.

#### Application of nano-formulations *in planta*

The prepared nano-formulations (Nano-1-Amp+Brij 35 and Nano-2-Amp+Brij 35), ampicillin solution (Amp), and water control (CK) were applied to cuticles from HLB-affected lemon trees with six replicates for the transcuticular movement assay. In addition, twelve 2-year-old grapefruit seedlings were treated with typical HLB symptoms by foliar spray six times at 2-week intervals. The treated seedlings were grown at 28°C ± 5°C in an insect-proof greenhouse. Five leaf samples were collected from each treatment at 30, 60, 90, 120, 180, and 240 days after initial treatment (DAT), and DNA was extracted for qPCR analysis.

### Genomic DNA extraction and qPCR analysis

Each leaf was rinsed three times with sterile water. Midribs were separated from the leaf samples and cut into pieces 1.0 to 2.0 mm in size. DNA was extracted from 0.1 g of the midrib tissue (fresh weight) using a Qiagen DNeasy Plant Mini Kit (Qiagen, Valencia, CA) according to the manufacturer’s protocol. qPCR was performed with primers and probes (HLBas, HLBr, and HLBp) specific for the detection of Las using an ABI PRISM 7500 sequence detection system (Applied Biosystems, Foster City, CA) [26]. A PCR reaction at a volume of 20 μl was composed of the following reagents: 300 nM (each) target primer (HLBas and HLBr), 150 nM target probe (HLBp), and 1 × TaqMan qPCR Mix (Applied Biosystems). The cycling conditions were 95°C for 20 s followed by 40 cycles at 95°C for 3 s and 60°C for 30 s. All reactions were performed in triplicate and each run contained one negative (DNA from healthy plant) and one positive (DNA from Las-infected plant) control.

### Data analysis

Variance analysis was carried out to individually compare the (i) cuticular isolations and (ii) characteristics from different citrus cultivars and HLB-affected plants as well as (iii) their physiochemical characterizations and (iv) the efficiency against Las bacterium of the nano formulations using the SAS/STAT procedure ANOVA. Differences among different treatments were assessed by Duncan's multiple range tests at p ≤ 0.05 (SAS V.9.1, SAS Institute, NC, USA).

## Results

### Isolation and characterizations of citrus cuticles

The isolation times presented in Table 2 indicate that isolation of cuticles was the most difficult from valencia orange compared to the other tested cultivars (lemon, grapefruit, and bitter orange). The results also show that the cuticle could not be isolated from any of the citrus cultivars in the largest disk (10 mm in diameter) with a low concentration of isolation buffer (1% pectinase and 0.1% cellulase). However, the isolation time (6.64 ± 4.16 d) in the high concentration of 4% pectinase and 0.4% cellulase was not significantly different from that in 2% pectinase and 0.2% cellulase (6.97 ± 4.66 d) (p = 0.578).

**Table 2:** Isolation time (days) of cuticles of four citrus cultivars using leaf disks of different sizes and different concentrations of pectinase and cellulase.

Concentration	Cultivar	Large (10mm)	Medium (7mm)	Small (4mm)
**4% pectinase + 0.4% cellulase**	Lemon	8.33 ± 0.29 b	5.17 ± 0.29 b	2.00 ± 0.00 b
	Grapefruit	8.67 ± 1.61 b	5.50 ± 0.50 b	2.00 ± 0.00 b
	Valencia	15.50 ± 0.00 a	11.00 ± 2.50 a	3.00 ± 0.29 a
	Orange	9.83 ± 1.15 b	6.50 ± 0.00 b	2.17 ± 0.00 b
**2% pectinase + 0.2% cellulase**	Lemon	8.17 ± 0.76 b	5.00 ± 0.50 c	1.67 ± 0.58 b
	Grapefruit	8.33 ± 0.29 b	6.67 ± 0.76 b	2.33 ± 0.29 ab
	Valencia	15.67 ± 0.29 a	15.00 ± 0.50 a	2.67 ± 0.29 a
	Orange	9.33 ± 3.62 b	6.50 ± 1.00 b	2.33 ± 0.29 ab
**1% pectinase + 0.1% cellulase**	Lemon	NI	5.83 ± 0.29 c	2.17 ± 0.29 b
	Grapefruit	NI	6.83 ± 0.58 b	2.50 ± 0.00 b
	Valencia	NI	15.50 ± 0.00 a	3.50 ± 0.00 a
	Orange	NI	7.00 ± 0.50 b	2.33 ± 0.29 b

All data were analyzed by Duncan’s multiple range test using SAS software package. Values denoted by the same letter (a or b) are not significantly different within the same treatment at p*≤* 0.05 level. NI indicates that cuticles could not be isolated from 10 mm disks in weak enzyme solution.

Notably, it was more difficult to isolate cuticles from HLB symptomatic leaves (Ct value = 26.30±1.36, isolation time = 14 d) compared to asymptomatic leaves (Ct value = 35.6±0.66, isolation time = 5.5 d). We also found that leaf extract from asymptomatic leaves had a 1.3-fold higher pectinase activity compared to symptomatic leaves (p = 0.034). Sucrose levels also significantly increased (approximately 35%) in symptomatic leaves (p = 0.045) (Table 3). The inhibitory zone diameter of cuticles isolated from asymptomatic leaves was 4.13 ± 2.27 mm, which was significantly higher than that from symptomatic leaves (p = 0.039) (Table 3).

**Table 3:** Comparison of several parameters between asymptomatic and symptomatic cuticles.

Parameters	Cuticles from asymptomatic leaves	Cuticles from symptomatic leaves
**Ct value**	35.6±0.66 a	26.30±1.36 b
**Isolation time (days)**	5.5	14
**Sucrose content (μg/g)**	328.46±53.13 b	427.56±24.92 a
**Fold change in pectinase activity** ^**c**^	1.29±0.06 a	0.97±0.07 b
**Inhibition zone diameter (mm)** ^**d**^	4.13±2.27 a	1.54±1.05 b

All data were analyzed by Duncan’s multiple range test using SAS software package. Different letters represent significant difference at a level of p *≤* 0.05.

^c^ Fold-change in pectinase activity of cuticles isolated from HLB-asymptomatic and symptomatic leaves.

^d^ Inhibitory zone diameter of cuticles treated with a 500 mg/L ampicillin solution.

### Identification of an optimal adjuvant for enhancing cuticle penetration

Seven of the eight candidate adjuvants, except NMP, enhanced cuticular permeability of lemon cuticles (Table 4). In addition, the permeability of Brij 35 was the highest among all of the tested adjuvants (~3.33-fold over CK). DMSO, Laurocapram, and Oleic acid also enhanced 4-NP permeability by 2.25-, 2.15- and 2.78-fold, respectively (Table 4). The other adjuvants, such as Brij 58, Brij 98, and Urea, enhanced 4-NP permeability by 1.63-, 1.33-, 1.80-fold, respectively (Table 4).

**Table 4:** Cuticular permeability of candidate adjuvants.

Classifications	Adjuvants	Permeability (10^−2^ mol.min^-1^)	Percentage (%)^e^	n-Fold ^f^
**Non-ionic**	**Brij 35**	1.841	232.91	3.33
	**Brij 58**	0.904	63.47	1.63
	**Brij 98**	0.995	79.93	1.80
**Anionic**	**Oleic acid**	1.538	178.22	2.78
**Sulfoxide**	**DMSO**	1.247	125.50	2.26
**Amide**	**NMP**	0.527	-4.70	0.95
	**Urea**	0.741	34.00	1.34
	**Laurocapram**	1.189	115.01	2.15
**Water control (CK)**		0.553	0	1.00

^e^ Percentage (%) was calculated as follows: $\text{Percentage in permeability}\;(\%)=\frac{Adjuvants-CK}{CK}\times 100$

^f^ n-Fold in permeability was calculated as follows: $\text{n}-\text{Fold}=\frac{Adjuvants}{CK}$.

### Physicochemical characterization and application of W/O nano-formulations

Two prepared W/O nanoemulsions (Nano-1-Amp and Nano-2-Amp) showed significantly different physicochemical properties (p = 0.041), but had excellent thermodynamic stability. The droplet size (5.26 ± 0.04 nm) and pH value (7.76 ± 0.03) of Nano-1-Amp was significantly smaller than those for Nano-2-Amp (droplet size = 94 ± 1.48 nm and pH value = 8.31 ± 0.05) (p = 0.013) (Table 5).

**Table 5:** Physiochemical characterizations of nanoemulsions.

Nanoemulsions	Droplet size (nm)	pH value	H/C	Cent.	Freeze-Thaw
**Nano-1-Amp**	5.26±0.04 A	7.76±0.03 A	√	√	√
**Nano-2-Amp**	94±1.48 B	8.31±0.05 B	√	√	√

H/C: Six heating-cooling cycles. Cent.: centrifugation at 3,000 rpm for 30min. Freeze-Thaw: Three freeze-thaw cycles. All data were analyzed by Duncan’s multiple range test using SAS software package. The different letters indicate a statistically significant difference (p ≤ 0.01).

**Fig 1:**
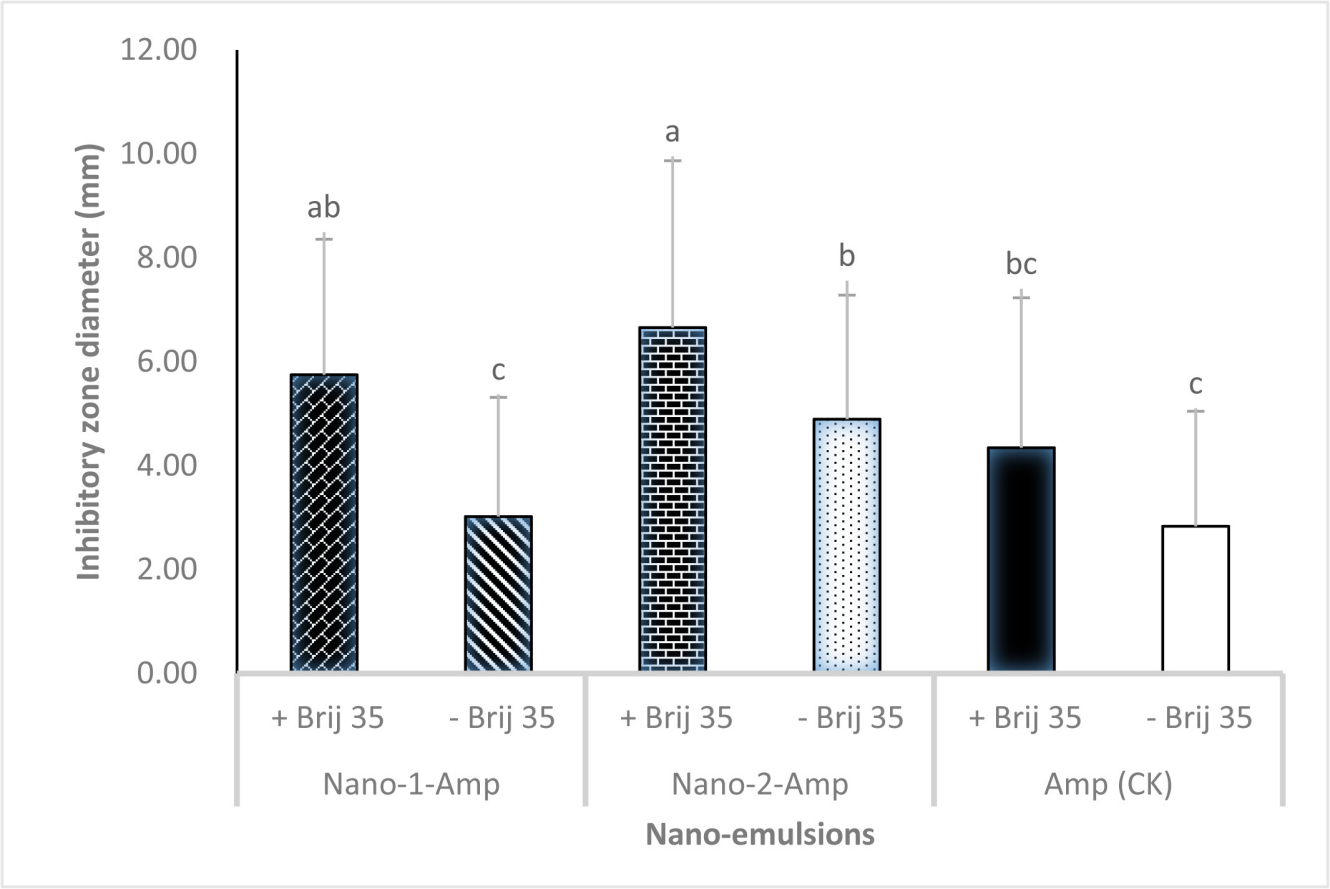
Inhibitory zone diameter of nano-formulations of Nano-1-Amp+Brij 35 and Nano-2-Amp+Brij 35. All data were analyzed by Duncan’s multiple range tests using SAS software package. Different letters represented significantly differences at the level of 0.05 (p *≤* 0.05).

Treatment with the novel nano-formulations containing Brij 35 (Nano-1-Amp+Brij 35 and Nano-2-Amp+Brij 35) resulted in inhibitory zone diameters of 5.75 mm and 6.66 mm, respectively, which were significantly larger than those from Brij 35 alone or nano-formulation without adjuvant (Nano-1-Amp and Nano-2-Amp) (p = 0.017) (Fig 1). Treatment with Amp alone resulted in the smallest inhibitory zone diameter (2.83 mm) (Fig 1). These results indicated that permeability through the isolated cuticles was greatly enhanced when Amp was loaded into nanoemulsions and coupled with Brij 35.

**Fig 2:**
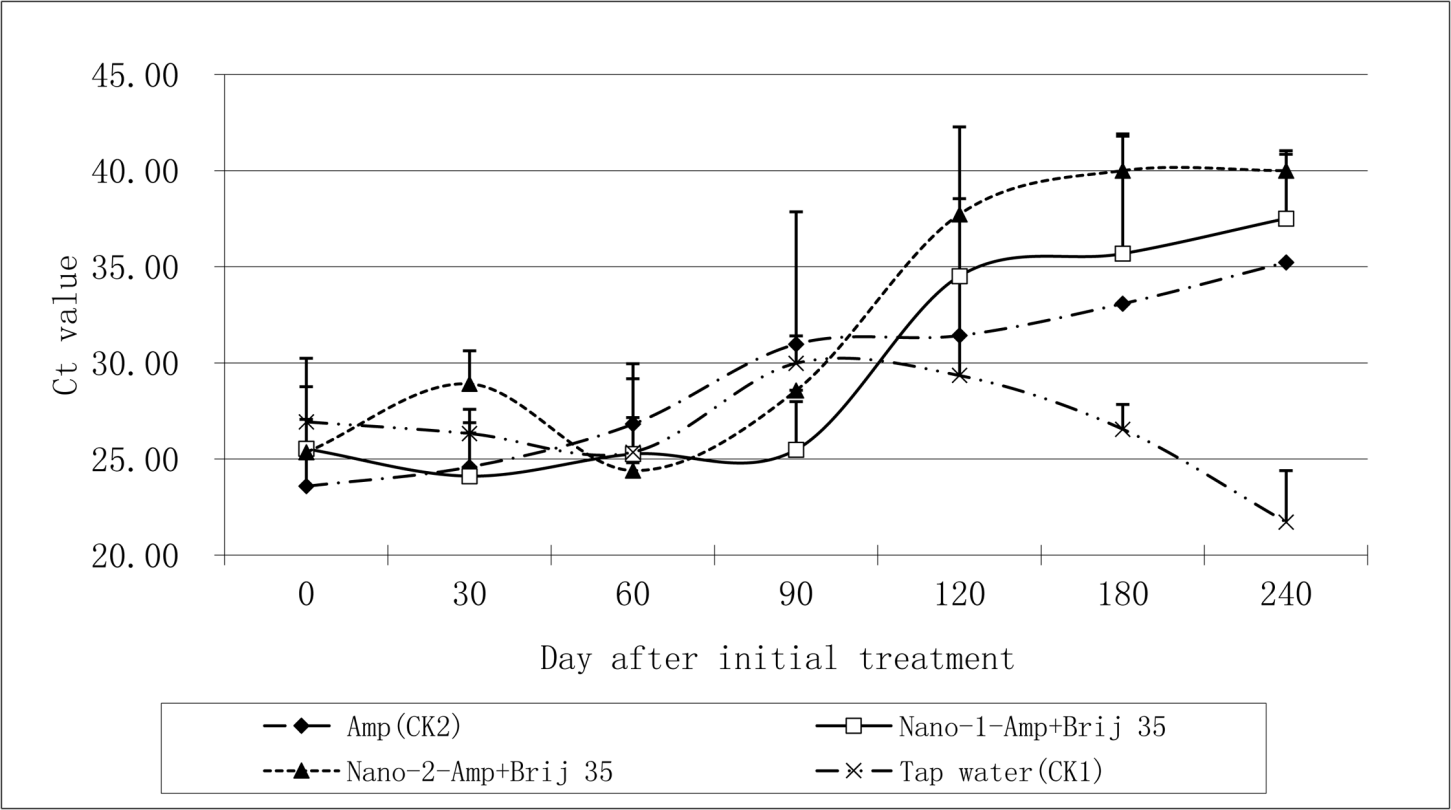
Ct value detected by qPCR in HLB-affected citrus after foliar spraying by nano-formulations.

**Fig 3:**
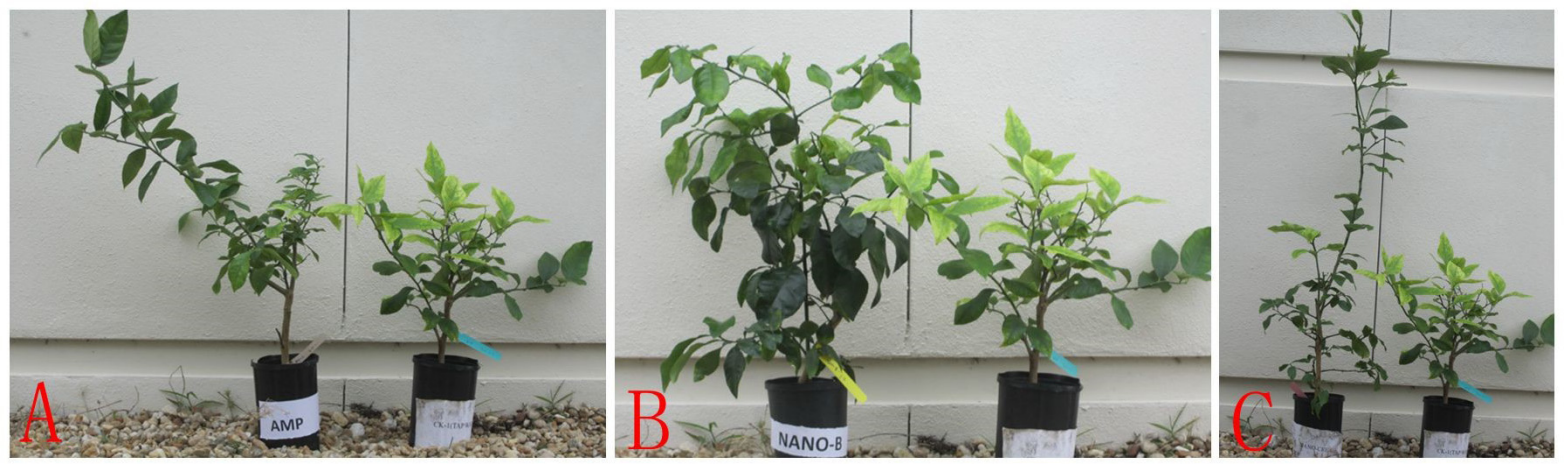
HLB-affected citrus treated by nano-formulations. (**A**): Amp (left) vs. tap water (CK) (right); (**B**): Nano-1-Amp+Brij 35 (left) vs. tap water (CK) (right); (**C**): Nano-2-Amp+Brij 35 (left) vs. tap water (CK) (right).

The results *in planta* showed that the optimized nano-formulations (Nano-1-Amp+Brij 35 and Nano-2-Amp+Brij 35) effectively reduced Las bacterial titers in HLB-affected citrus for 8 and 4 months, respectively (Fig 2). Foliar application of Amp without loading into nanoemulsions and in absence of Brij 35 (CK) also suppressed Las, but the bacterial titers in these treatments were higher than those observed in the optimized W/O nano-formulations at 4 to 8 months after initial treatment (Fig 2). All treated plants became asymptomatic and had lower bacterial titers at 240 days after initial treatment, except those that were treated with CK (Figs 2 and 3).

## Discussion

Huanglongbing is a serious citrus disease occurring worldwide. Since the causative Las bacterium resides in the phloem, a significant challenge exists in the delivery of effective antimicrobial treatments. In this study, economical water in oil (W/O) nano-formulations (Nano-1-Amp+Brij 35 and Nano-2-Amp+Brij 35) using the ideal adjuvant Brij 35 and W/O nanoemulsion were optimized and developed for delivering ampicillin through the cuticle into citrus phloem by foliar spray.

Ampicillin is one of the most effective antimicrobial compounds against Las bacterium and exhibits the lowest phytotoxicity to citrus [12, 36]. It is taken up rapidly and degraded easily by plants, and therefore its use for treating and curing Las-infected citrus trees would provide a great commercial value. However, development and/or refinement of application techniques are needed prior to commercial application. Due to public concerns on the emergence of antibiotic-resistant bacteria and the potential side effects on humans, the application of ampicillin to crops has not yet been approved in commercial groves by the Environmental Protection Agency (EPA) or other regulatory agencies. Therefore, in this study ampicillin was used as an antimicrobial test compound to assess the efficacy of a nanoemulsion delivery system in the treatment of citrus plants in a greenhouse. When Amp was loaded into the W/O nanoemulsions and prepared as optimized nano-formulations coupled with a screened adjuvant (Brij 35) ideal for enhancing penetration, the prepared nano-formulations (Nano-1-Amp+Brij 35 and Nano-2-Amp+Brij 35) significantly increased Amp penetration through the cuticle. The nano-formulation showed larger inhibitory diameter zones in an *in vitro* bioassay and more effectively suppressed or eliminated Las bacterium in a *in planta* assay compared to Amp or Brij 35 alone (Figs 1 and 2). Compound penetration is different based on the specific formulation of various nanoemulsions [37–39]. We found that Nano-2-Amp+Brij 35 eliminated Las bacterium from HLB-affected citrus plants more effectively than Nano-Amp-1+Brij 35 (Fig 2). This difference may have resulted from a difference in Amp penetration in the cuticle. Although the constituents of these two nanoemulsions were exactly the same, the methods of their preparation were different. This observation underscores the postulate that fine physiochemical properties of the nanoemulsions, such as droplet size and pH value, resulted in different delivery efficiencies into phloem. For Nano-2-Amp, the ampicillin was immerged into the nanoemulsions as the first step of the nanoemulsion preparation, whereas for Nano-1-Amp, it was added during the second step. Although the droplet size of nanoemulsions (Nano-1-Amp and Nano-2-Amp) was 5.26 ± 0.04 nm and 94 ± 1.48 nm, respectively, both nanoemulsions had a low surface tension and low interface tension of the droplets due to the small droplet size (≤ 100 nm) [38, 40, 41]. Moreover, the nanoemulsions increase the dispersion and wettability of the formulations, offering a large specific surface area and increased affinity for enhancing the permeation of Amp through the citrus leaf.

Cuticle isolation was dependent on citrus cultivars and Las bacterial infection. The valencia orange cuticle was the most difficult to isolate compared to lemon, grapefruit, and bitter orange (Table 2). Cuticles were also more difficult to isolate from HLB-symptomatic leaves than those from asymptomatic leaves, which was due to the accumulated starch in HLB-affected citrus leaves and the inhibition of pectinase by sucrose in vitro[42–45]. In the transcuticular movement assay, the inhibitory zone diameter of asymptomatic cuticles was 4.13 ± 2.27 mm, which was significantly larger than that of symptomatic cuticles (Table 3). Therefore, it is reasonable to hypothesize that Las bacterium affects citrus cuticle properties, which results in the transcuticular permeability variation. However, the mechanism remains to be elucidated.

Brij 35 was found to induce 3.33-fold greater cuticle permeability than control, which was the highest of the 8 adjuvants tested (Table 4). Brij 35 disrupts the lipid arrangement and increases water content in waxes [46, 47]. The lipophilic domain becomes fluidized and promotes the diffusion of adjuvants. This perturbation likely fluidizes the lipids in the cuticle and, consequently, allows complex molecules to pass through [48]. Brij 35 has been previously used to deliver pesticides and fertilizers through the plant cuticle [49, 50]. Therefore, Brij 35 is a very promising agent for the delivery of antimicrobials to combat HLB disease.

The eight adjuvants tested in this study can be divided into four types: non-ionics, anionics, amides, and sulfoxides. The chemical properties among each type may also be different, such as hydrophobicity, polyethylene oxide (EO) chain length, and hydrophilic lipophilic balance value [48]. The eight adjuvants demonstrated observable differences in their ability to affect cuticular permeability (Table 4). DMSO, oleic acid, and laurocapram were also effective adjuvants (Table 4). In a previous report, DMSO did not have a significant effect on the combination of penicillin G and streptomycin for eliminating or suppressing Las bacterium (p = 0.823) [11]. Oleic acid and laurocapram are incompatible with aqueous solutions of ampicillin due to their insolubility [51, 52]. Although other adjuvants (Brij 58, Brij 98, and Urea) enhanced cuticular permeability, their effectiveness was significantly lower compared to Brij 35 (p = 0.024) (Table 4). Therefore, these adjuvants were not pursued further as components of an antimicrobial delivery process in this study. NMP was the only adjuvant that did not enhance cuticular permeability.

In summary, our optimized nanoemulsions were more efficient, stable, and economical for controlling HLB disease by foliar spray. Furthermore, unlike other traditional formulations, the organic solvent ethyl acetate, which is harmful to the environment, was removed through evaporation during the preparation process. Traditional delivery methods, such as foliar spray or bark painting, do not provide adequate penetration of chemicals into the phloem. Our environmentally friendly nanoemulsions provide a more effective delivery system of antimicrobial compounds targeted to the citrus phloem via foliar spray as an approach to control citrus HLB.
